# 

**Published:** 1906-08

**Authors:** 


Criminal Responsibility of the Insane.

VOL. XXII. AUGUST, 1906. No. 2.

AUG. 10 1906

LIBRARY INDEXED S.G.O.

### TEXAS MEDICAL JOURNAL

"RED BACK"

PUBLISHED AT AUSTIN, TEXAS, BY

F. E. DANIEL, M. D., - - - Proprietor

PRINTED BY THE VON BOECKMANN-JONES CO.

Subscription, $1.00 a Year, in Advance. - - - Single Copies, 10 Cents

Entered at the Postoffice at Austin, Texas, as Second-class Mail Matter

